# Integrating Data and Resources on Neglected Tropical Diseases for Better Planning: The NTD Mapping Tool (NTDmap.org)

**DOI:** 10.1371/journal.pntd.0003400

**Published:** 2015-02-05

**Authors:** Rebecca M. Flueckiger, Birgit Nikolay, Huub C. Gelderblom, Jennifer L. Smith, Danny Haddad, Wesley Tack, Guy Hendrickx, David Addiss, Jorge Cano, Danny R. Hatcher, Adrian Hopkins, Rachel L. Pullan, Alex Pavluck, Eric Ottesen, Simon J. Brooker

**Affiliations:** 1 NTD Support Center, Task Force for Global Health, Emory University, Atlanta, Georgia, United States of America; 2 International Trachoma Initiative, Task Force for Global Health, Emory University, Atlanta, Georgia, United States of America; 3 London School of Hygiene & Tropical Medicine, London, United Kingdom; 4 Emory Eye Center, Emory University, Atlanta, Georgia, United States of America; 5 Avia-GIS, Zoersel, Belgium; 6 Children without Worms, Task Force for Global Health, Emory University, Atlanta, Georgia, United States of America; 7 Environmental Systems Research Institute, Redlands, California, United States of America; 8 The Mectizan Donation Program, Task Force for Global Health, Emory University, Atlanta, Georgia, United States of America; 9 RTI International, Washington D.C., United States of America; University of Yaoundé I, CAMEROON

## Introduction

Neglected tropical diseases (NTDs) affect more than 1,000,000,000 poor and marginalized people worldwide [1]. NTDs are caused by diverse pathogens with differing modes of transmission and a range of vectors and intermediate hosts, which have their own ecological peculiarities. While there is considerable overlap in the geographical distribution of different NTDs at a national level [1], epidemiological differences of individual NTDs give rise to marked geographical variation at local levels. Since cost-effectiveness of intervention is greatest when targeted to areas having a high burden of multiple diseases, maps of the distribution of the different NTDs are essential for planning and implementing NTD interventions, as well as for providing visualization of program progress, so important for advocacy. In recent years there have been concerted, and very successful, efforts to develop detailed information resources on the geographical distribution of different NTDs (Table 1).

**Table 1:** Currently available resources on the geographical distribution of NTDs.

**Mapping initiative**	**NTDs^1^**	**Sources of data**	**Description of platform**	**Website**
WHO preventive chemotherapy (PCT) databank	LF, STH, Sch, Tr	Country PCT data as officially reported to WHO.	Static country profiles including estimates of populations at risk, population requiring PCT, and program coverage. Country level tabular summaries are downloadable. Interactive LF mapping by country.	http://www.who.int/neglected_diseases/preventive_chemotherapy/databank/en/index.html
African Programme for Onchocerciasis Control (APOC)	Oncho, Loa loa	Field surveys using REMO or RAPLOA.	Static, downloadable country level maps of onchocerciasis and loa loa distribution, identifying areas requiring community-directed treatment with ivermectin.	http://www.who.int/apoc/countries/en/
Global Atlas of Helminth Infection [10]	STH, Sch, LF	Structured searches of published and unpublished sources.	Static, downloadable country maps of survey data, predictive prevalence, and districts requiring MDA. Data available to download for selected countries and progress ongoing.	http://www.thiswormyworld.org
Global Atlas of Trachoma [7]	Tr	Structured searches of literature and unpublished data from program managers.	Static, downloadable country level maps of the distribution of active trachoma in children under one to nine years and trichiasis in adults ≥ 15 years.	www.trachomaatlas.org
International Coalition for Trachoma Control	Tr	SAFE activities as reported by NGOs.	Static, downloadable country level maps of implementation of SAFE components.	www.trachomacoalition.org
Global NTD database [12]	STH, Sch	Structured searches of published and unpublished sources.	Tabular form, including information on location, year, ages, and prevalence. No mapping platform currently available.	www.gntd.org

^1^LF = lymphatic filariasis, STH = soil-transmitted helminths; Sch = schistosomiasis; Oncho = onchocerciasis; Tr = trachoma; SAFE = surgery, antibiotics, facial cleanliness, and environmental improvement, NGO = nongovernmental organization, REMO = rapid epidemiological mapping of onchocerciasis, RAPLOA = rapid assessment procedure for Loa loa.

An important element of targeted NTD intervention is the delivery of mass drug administration (MDA) for treating the five major “preventive chemotherapy” NTDs, including lymphatic filariasis (LF), onchocerciasis, schistosomiasis, soil-transmitted helminths (STH), and trachoma [2]. MDAs targeting these NTDs are implemented alongside improvements in water and sanitation and hygienic behavior, as well as vector control. To help galvanize such global health efforts, the World Health Organization (WHO) and the NTD community defined targets to be achieved by 2020 and strategies to reach these targets (Table 2). As countries make progress towards the 2020 goals with an ever-increasing amount of data being collected, it is important to develop readily accessible tools that policymakers and program staff and partners can use to access, visualize, and compare data.

**Table 2:** The five main NTDs and the drugs and strategies used to target them programmatically.

Disease	Drug(s)	Strategy	Target
**Lymphatic filariasis**	albendazole/ivermectin or albendazole/DEC	MDA* + vector control (mainly bed nets)	Global elimination by 2020 [13, 14]^i^
**Onchocerciasis**	ivermectin	MDA + vector control [15, 16]	Regional and country elimination by 2025 [16],^ii^
**Schistosomiasis**	praziquantel	MDA + clean water and adequate sanitation, hygiene education, snail control [2, 17]	Control morbidity by 2020 and elimination by 2025 [14, 17]
**Soil-transmitted helminths**	albendazole or mebendazole	MDA + clean water, sanitary latrines, hygiene education [18, 19]	Control^iii^
**Trachoma**	azithromycin	MDA + surgery to correct trichiasis + facial cleanliness + environmental improvement (SAFE) [20]	Global elimination of blinding trachoma by 2020 [14, 20],^iv^

^i^Prevalence of infection with *Wuchereria bancrofti*, *Brugia malayi*, or *Brugia timori* less than target thresholds in all endemic areas in all countriessite. Interrupt transmission and reduce the at-risk population to zero [13]

^ii^Prevalence of infection with *Onchocerca volvulus* less than target threshold in children less than ten years old, and prevalence of infective larvae in *Simulium* flies less than target threshold. Verified after a minimum period of 3–5 consecutive years of adequate postintervention surveillance

^iii^Control is the elimination of infections of moderate and high intensity (Ascarsis lumbricoides >5,000 epg, Trichuris trichiura >1,000 epg, Necator americanus or Ancylostoma duodenale >2,000 epg) [19]

^iv^Incidence of less than one case of trichiasis per 1,000 population and less than 5% of children between the ages of one and nine years old having active disease (TF) in each endemic country. Verified after a minimum period of three consecutive years of adequate postintervention surveillance

*MDA—Mass Drug Administration—can target entire at-risk communities (e.g., for LF or oncho) or segments of the population (e.g., school-age children for STH)

In this *Innovation to Application* article, we describe the creation of an innovative NTD mapping tool (www.ntdmap.org) developed by a consortium of research and program partners for use particularly by program implementers. Its functionality and accessibility have been designed specifically to meet the needs of national programs and international partners. This tool provides an online resource allowing users to visualize and manipulate geographical data on a range of variables for the planning and managing of integrated NTD programs.

## Current Mapping Resources

Sharing data is challenging not only because of the complexity of the data themselves but also from the sheer volume of information. In an effort to meet these challenges, the NTD community has developed a range of resources that display epidemiological and treatment data in the form of maps (Table 1). Data are typically displayed as static maps that can be freely downloaded and reused. Similar mapping initiatives have also been undertaken for other diseases, including malaria [3], human trypanosomiasis [4], and leishmaniasis [5].

While these disease-specific mapping projects are extremely helpful for visualizing predefined projects, they are of limited usefulness for integrated program planning, primarily because of the inability to customize maps through overlaying multiple variables highlighting cross-disease opportunities. These resources are also time-consuming to maintain, with data needing to be managed manually for each tool. In the rapidly changing NTD environment, the utility of maps erodes quickly with time. To become a useful operational resource, maps must be routinely updated. For NTDs, this need has been addressed in the development of an NTD mapping tool capable of integrating available epidemiological and programmatic NTD data into a single resource, which is both dynamic and easy to use.

## Developing the Tool

A first step in developing the tool was a stocktaking exercise of existing mapping platforms and an end-user survey to assess the needs of the tool’s intended audiences. The exercise included a “state-of-the-art” review of existing mapping applications, undertaken by independent consultants, Avia-GIS (http://www.thiswormyworld.org/about/gahi-reports). Each platform was reviewed for content, quality, and functionalities offered. Results show that the main functionalities included were (i) ability to add different map layers, (ii) query the data underpinning each map, (iii) zoom and pan in the maps, and (iv) print and save maps.

**Fig 1:**
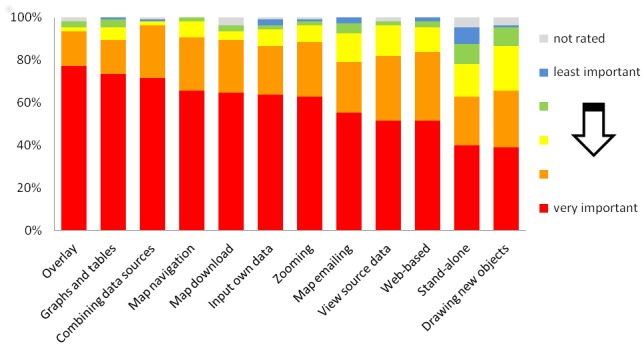
Ratings of functionality of mapping tool functions, based on an end-user survey of 105 participants from 45 countries, 2012.

**Fig 2:**
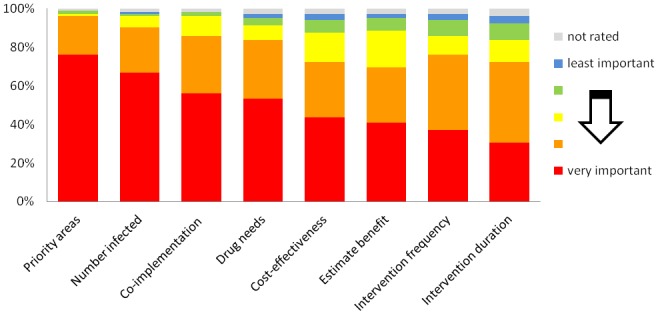
Ratings of applications of a mapping tool, based on an end-user survey of 105 participants from 45 countries, 2012.

As the goal of this project is to provide a single tool with functionalities that are particularly useful for the planning and evaluation of NTD programs, the second step of the development was a needs assessment among staff from NTD control programs, international agencies, and funding agencies. A questionnaire asking participants about potential functions of an NTD mapping tool and the main uses to which it would be applied was developed. The questionnaire was piloted in Kenya, and a final version was distributed at international NTD meetings in 2012 and was additionally made available online. In total, 105 participants from 45 countries took part in the survey. Figs. 1 and 2 present the main survey results and indicate that the functions given the highest priority were the ability to (i) overlay map layers, (ii) combine data sources, (iii) navigate to specific areas, (iv) download data, and (v) draw tables.

**Fig 3:**
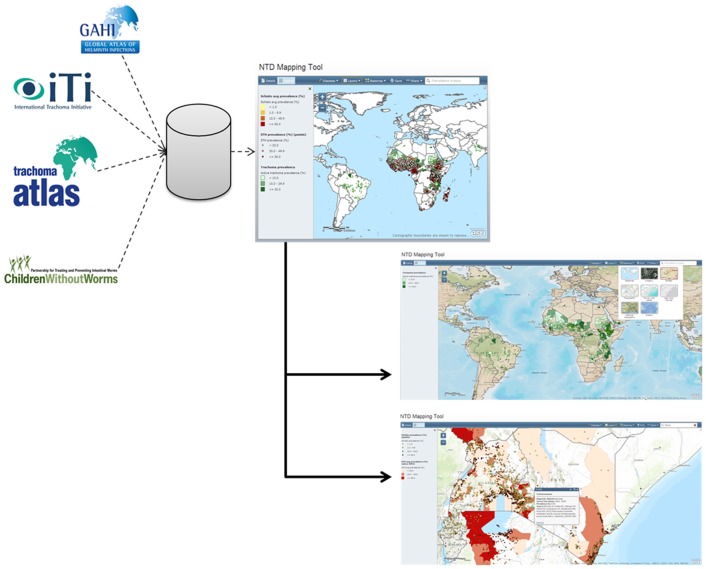
Linking existing databases through a mapping server allows for geographic display of tabular data.

Finally, a series of meetings were held among partners in March, 2013. These meetings established the minimum criteria for the NTD mapping tool (Table 3). To assist in the technical development of the tool, Esri, the company which makes the Geographic Information System software ArcGIS (www.esri.com), provided high-level software expertise. The technical development of the tool included linking Structured Query Language (SQL) databases from multiple partners such as the Global Atlas of Helminth Infections (GAHI) [6], Global Atlas of Trachoma [7], International Trachoma Initiative (ITI) and Children Without Worms (CWW) drug donation databases, and maps of access to water and sanitation [8]. These datasets were linked based on commonly shared health district level unique identifiers through cloud-based servers to an Esri-powered Geographic Information System (GIS) server which pushes the information up to the website as GIS layers (Fig. 3).

**Table 3:** NTDmap.org minimum criteria.

**Categories**	**Criteria**
Epidemiological uses	1) Determining current distribution of each NTD 2) Identifying coendemicity of different NTDs 3) Identifying areas without survey data
Programmatic uses	1) Assessing intervention coverage 2) Identifying intervention gaps 3) Forecasting future intervention needs 4) Supporting evaluation and surveillance efforts
Data to include	1) Endemicity status: known to be endemic above threshold for MDA; known to be nonendemic above threshold for MDA 2) Treatment data: whether MDA is ongoing (yes/no); whether adequate MDA has been achieved, based on NTD-specific threshold (yes/no) 3) WASH information
Administration	1) Information will be provided from the NTD-specific atlases on a quarterly basis 2) Information will be pulled from existing databases, leaving quality control in the hands of the original data managers

A beta-version of the tool was piloted at an NTD training course in Nairobi in May 2013 (http://www.thiswormyworld.org/training/modern-tools-for-ntd-control-programmes). Feedback received included the ability to adjust the order of layers, inclusion of administrative boundary layer, ability to query the source of data, and the ability to change the colors of layers. A second version of the tool was then released to selected partners on September 1, 2013 for additional feedback. The process of requesting feedback from both field managers and partner organizations ensured the usefulness of the tool to the broader NTD community. The completed tool was launched at the NTD Non-Governmental Development Organization (NGDO) Network (NNN) meeting in Brighton, United Kingdom, on September 19, 2013.

## Features of the Tool

**Fig 4:**
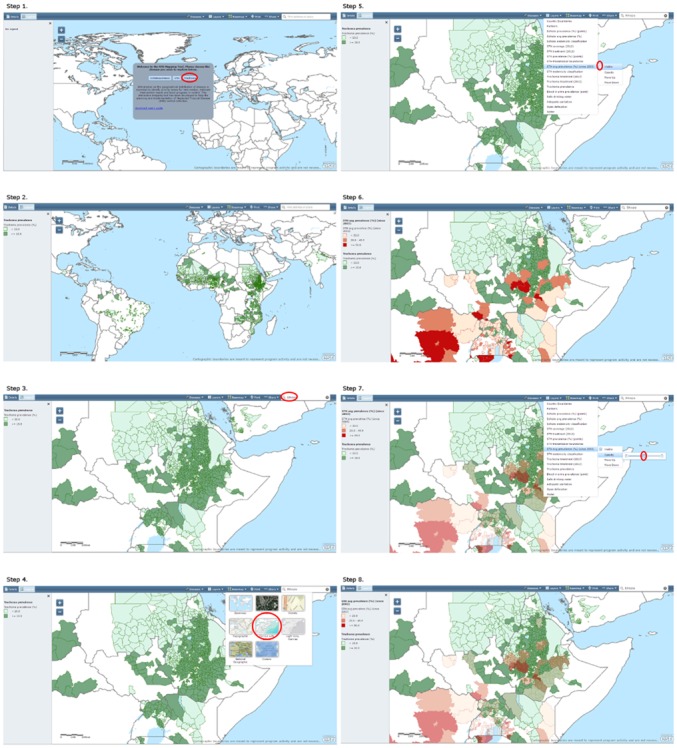
Practical use of NTDmap.org, trachoma, and STH in Ethiopia.

Linking data from multiple partners allows users to view information on one disease or a combination of diseases on the same map (Fig. 3). Additionally, the user can add layers showing road networks, satellite imagery, and topography. The ability to adjust opacity of each layer allows for viewing multiple variables at one time. The tool contains pop-up windows with information referencing the source of the displayed data as well as the methods used in data collection, a feature that ensures openness among partners, facilitates data cross-checking among the multiple reporting mechanisms, and provides valuable information for programmatic decision making. The NTD Mapping Tool harnesses the power of the individual NTD mapping resources allowing users to combine information together and generate customized maps that meet their specific needs. For example, an NTD manager in Ethiopia can visually depict the distribution of both STH and trachoma on one map and thus help guide resources and strategies for a coordinated approach to MDA. The manager may also view where MDA occurred last year for these diseases, highlighting progress and treatment gaps (Fig. 4). This sharing of information in a geographic platform promotes integrated NTD work in country, as the web-based tool allows partners to generate these customized maps without the need to acquire specific GIS software and skills.

## Lessons Learned and Future Developments

Use of the tool to date has already resulted in a number of challenges identified and lessons learned (Box 1). First, differences in the data, including date of collection and survey methodology, hinder direct comparison of analyzed survey data. For example, a recent systematic review of STH diagnostics has highlighted the wide differences in the reliability of available STH diagnostic methods [9], which will reduce the comparability of data—a challenge faced by all mapping resources. This could be addressed by scoring the quality of included data based on age of survey, study population, sample size, sampling method, and diagnostic tests. Guidelines for such scoring are currently under development by WHO and will be applied to standardize disease mapping when available. To assist in understanding the time frame of the data, a time stamp has now been included in the tool, which will allow users to see when the information on the map was last updated and downloaded, thereby creating openness and consistency within the system.

**Box 1. Advantages and Disadvantages of www.NTDmap.org**

**Advantages**

- Allows users to generate customized maps without the need to acquire specific GIS software and skills.
- Provides a platform to view data on different NTDs and overlay distributions.
- Enables user to generate maps for printing and presentations.

**Disadvantages**

- Geographic administrative boundaries change frequently, over time this can cause confusion when examining data in a map format.
- The current design of the tool does not allow users to add their own data. Future releases will have this capability.
- A standard internet connection is necessary to use the tool. This is a challenge in some of the areas that would most benefit from a tool like this. Future releases will include offline capability.

Second, differences in map boundaries can be a major source of potential confusion. Occasionally, programs use political administrative boundaries when implementing or surveying for one disease and different health district boundaries for another. This creates difficulties when overlaying the information from various diseases. As standardized protocols and data collection methods are adopted, the tool will be refined and adapted to reflect these changes. The Mapping Tool presents treatment coverage provided by the drug donation programs (The International Trachoma Initiative and Children Without Worms) for the different NTDs at the subnational (typically district) level, whereas WHO typically reports coverage at the national level. Better understanding of the relationship between these two sources would be useful for filling knowledge gaps on treatment coverage. Third, the NTD Mapping Tool includes data on STH, schistosomiasis, and trachoma, as well as health district maps of access to improved water and sanitation. It is envisioned that in the future, data on LF [10] and onchocerciasis [11] will be included, increasing the potential combinations to overlap treatment and disease.

A second phase of development on this tool is underway. In the upcoming release, the technical features will be enhanced. This release will include an offline component where users with limited internet access can build their own maps. Users will also have the ability to input their own data into the offline maps, allowing the visualization of private data in the context of the publicly available data included in the tool. Future work will additionally investigate how best to make the data included in the tool available for download; at present, data on STH, schistosomiasis, LF, and Water Sanitation and Hygiene (WASH) can be downloaded from the Global Atlas of Helminth Infection (www.thiswormyworld.org). Readers interested in the details of these data can consult this resource and contact this project.

In conclusion, the NTD Mapping Tool provides an important new tool for the planning, implementation and evaluation of NTD control activities. The tool is simple and intuitive to use, with minimal requirements placed on the users. The development of the tool has brought together partners working on different NTDs and has involved extensive consultation with the global NTD community. Future roll-out of the tool will identify further needs which will guide subsequent revision. The experience of developing the NTD Mapping Tool provides a powerful example of how integrating data, expertise, and resources can provide new resources and tools to help improve decision making in, and implementation of, integrated health programs.
